# Epitome of Diseases of the Skin

**Published:** 1885-12

**Authors:** 


**Epitome of Diseases of the Skin.** Being an abstract of a course of lectures by Louis Duhring, M. D. Reported by HENRY WILE, M. D. 12 mo.; cloth; price, 60c. Philadelphia: J. B. Lippincott Co. 1886.

This little work contains the abstracts of Duhring's lectures, originally published in the *Medical News.* They are hardly worth publication in book form. Those who have the admirable and complete work of Duhring do not need this, and those who have not, either student or practitioner, had better buy a more complete work.

